# Potential cross-species transmission of highly pathogenic avian influenza H5 subtype (HPAI H5) viruses to humans calls for the development of H5-specific and universal influenza vaccines

**DOI:** 10.1038/s41421-023-00571-x

**Published:** 2023-06-16

**Authors:** Pan Huang, Lujia Sun, Jinhao Li, Qingyi Wu, Nima Rezaei, Shibo Jiang, Chungen Pan

**Affiliations:** 1 Laboratory of Molecular Virology & Immunology, Technology Innovation Center, Haid Research Institute, Guangdong Haid Group Co., Ltd., Guangzhou, Guangdong China; 2 Key Laboratory of Medical Molecular Virology (MOE/NHC/CAMS), Shanghai Institute of Infectious Disease and Biosecurity, School of Basic Medical Sciences, Fudan University, Shanghai, China; 3 Research Center for Immunodeficiencies, Children’s Medical Center, Tehran University of Medical Sciences, Tehran, Iran

**Keywords:** Immunology, Molecular biology

## Abstract

In recent years, highly pathogenic avian influenza H5 subtype (HPAI H5) viruses have been prevalent around the world in both avian and mammalian species, causing serious economic losses to farmers. HPAI H5 infections of zoonotic origin also pose a threat to human health. Upon evaluating the global distribution of HPAI H5 viruses from 2019 to 2022, we found that the dominant strain of HPAI H5 rapidly changed from H5N8 to H5N1. A comparison of HA sequences from human- and avian-derived HPAI H5 viruses indicated high homology within the same subtype of viruses. Moreover, amino acid residues 137A, 192I, and 193R in the receptor-binding domain of HA1 were the key mutation sites for human infection in the current HPAI H5 subtype viruses. The recent rapid transmission of H5N1 HPAI in minks may result in the further evolution of the virus in mammals, thereby causing cross-species transmission to humans in the near future. This potential cross-species transmission calls for the development of an H5-specific influenza vaccine, as well as a universal influenza vaccine able to provide protection against a broad range of influenza strains.

## Introduction

Avian influenza is an infectious disease that affects poultry and wildfowl. It is caused by highly pathogenic avian influenza (HPAI) or low pathogenic avian influenza (LPAI) viruses, which belong to the Orthomyxoviridae family and have a single-stranded negative-sense RNA genome. Avian influenza viruses (AIVs) are mainly classified on the basis of their surface proteins, hemagglutinin (HA) and neuraminidase (NA). HA protein on the surface of the virion, the main antigenic site in vaccine design, causes erythrocyte agglutination in vitro and in vivo^1^. Over the years, outbreaks of HPAI H5 subtype viruses in poultry have caused huge economic losses to the farming industry. In 2022, more than 25 million poultry and wild birds were infected with HPAI H5 worldwide, resulting in 5.28 million deaths (https://wahis.woah.org/). Recently, HPAI H5 has caused more sporadic cases, or even outbreaks, in mammals, including minks, otters, foxes, and sea lions^2–4^. With possible further mutations in avian and mammalian species, HPAI H5 has a strong potential to cause human infection and trigger a global pandemic. Therefore, it is essential to develop an H5-specific vaccine, as well as a universal influenza vaccine, to fully cover a broad range of influenza strains.

### Global distribution of HPAI H5 viruses

H5N1 was the first strain isolated among the HPAI H5 viruses in Scotland in 1959, and it was shown to infect a variety of avians^5^. In 1997, HPAI H5N1 (Gs/GD/96) emerged in China and it was first confirmed to infect humans^6^. In 2000, H5N1 broke out among poultry in several countries, including the Netherlands, Vietnam, Indonesia, and Thailand^7^. A few years later (after 2005), H5N1 further spread to poultry in Europe and Africa^8,9^. Owing to homologous recombination among influenza strains in poultry, other non-N1 recombinant AIVs strains, such as H5N2, H5N6, and H5N8, have emerged in many countries. To classify H5 subtype AIVs, the HA gene was selected by the WHO/OIE/FAO H5N1 Evolution Working Group to divide AIVs into diffident clades based on the similarity of HA nucleic acid sequences. Each distinct clade was determined to have an average distance > 1.5% from other clades^10^. From 2013 to 2019, HPAI viruses of subclades 2.3.2.1 and 2.3.4.4 began to spread around the world^11–16^. HPAI H5 subclade 2.3.4.4 was first detected in domestic ducks in China^17,18^ and was further divided into 8 subclades, 2.3.4.4a to 2.3.4.4h^19^.

**Fig. 1:**
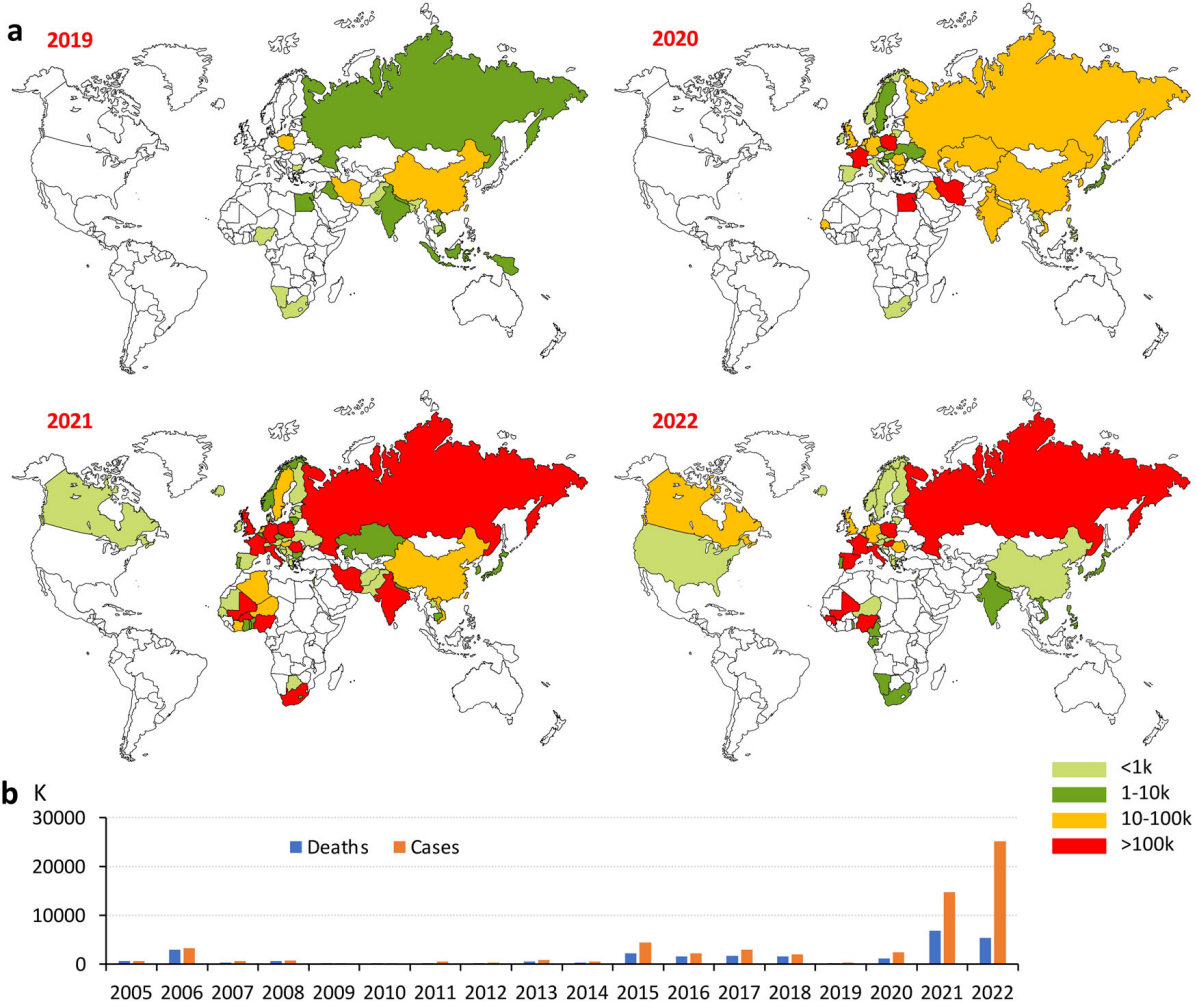
Global distribution of HPAI H5 viruses is shown by avian cases and deaths from 2019 to 2022 (https://wahis.woah.org/#/dashboards/qd-dashboard). Data up to January 2023 are included. **a** Distribution of HPAI H5 viruses between 2019 and 2022 based on the number of infections. **b** Number of avian infections and deaths caused by HPAI H5 viruses from 2005 to 2022.

**Fig. 2:**
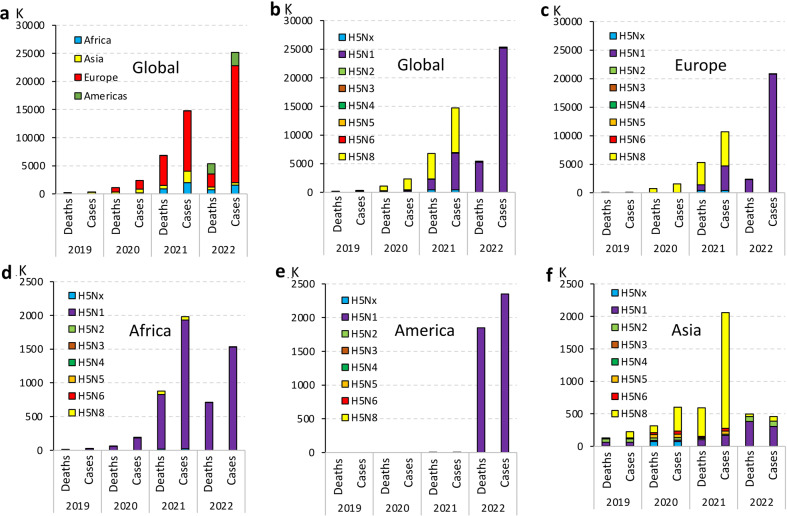
Global distribution of HPAI H5 avian infections from 2019 to 2022. **a** Distribution of HPAI H5 in Asia, Europe, Africa, and the Americas. **b** Global distribution of each subtype of HPAI H5. **c**–**f** Continent-specific distribution of each subtype of HPAI H5. Raw data for avian cases and deaths were taken from WAHIS (https://wahis.woah.org/#/dashboards/qd-dashboard). Data are included up to January 2023.

From 2019 to 2022, HPAI H5 viruses have been circulating among avian populations in Europe, Africa, and Asia^20–24^, resulting in a significant increase in global avian cases from 0.343 to 25.19 million (Fig. 1). Europe has become the primary site of spread accounting for 82.7% of avian cases and 43.9% of deaths globally in 2022 (Fig. 2a). Notably, the main HPAI subtype virus causing global epidemics gradually changed from H5N8 to H5N1 between 2019 and 2022 (Fig. 2b). For example, the epidemic of HPAI viruses in Europe was dominated by H5N8 from 2019 to 2021. However, it changed to H5N1 in 2022. During this year, infections and mortality rates caused by H5N1 accounted for ~99.9% among all HPAI H5 viruses in the same period (Fig. 2c). Since 2019, the H5N1 subtype has been dominant in Africa and the Americas, accounting for more than 99.9% of cases (Fig. 2d, e). Similar to Europe, H5N8 was the main HPAI subtype in Asia from 2019 to 2021, but it also changed to H5N1 in 2022. In 2022, the H5N1 subtype accounted for 67.4% of infections and 76.3% of mortalities among all H5 subtypes in Asia (Fig. 2f).

### Human infections with HPAI H5 viruses

**Fig. 3:**
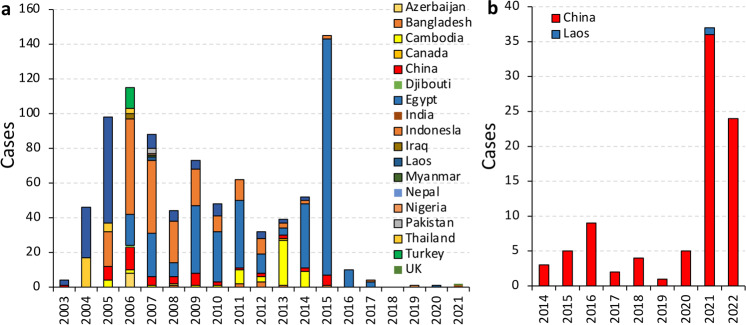
Global number of human cases of H5N1 and H5N8 infection. **a** Global number of human cases of H5N1 infection from 2003 to 2021 (https://www.who.int/publications/m/item/cumulative-number-of-confirmed-human-cases-for-avian-influenza-a(h5n1)-reported-to-who-2003-2023-3-march-2023). **b** Global number of human cases of H5N6 infection from 2014 to 2022 (https://search.fresh.gov.hk/chp/sc/search_result.php?q=influenza+virus&fq_yr=2023&fq_ct=&fq_ft=&sort=&page=1).

The current global epidemic of HPAI H5 mainly involves three subtypes: H5N1, H5N8, and H5N6. Indeed, the widespread epidemic of AIVs among wild birds increases the risk of infection for poultry and other avians. However, it is generally believed that human infection with HPAI H5 viruses is closely related to outbreaks in poultry and wild birds, and according to the WHO, 864 human cases of H5N1 infection have been reported worldwide, resulting in 456 deaths from 2014 to 2021(Fig. 3a). It was previously believed that only cumulative mutations of AIVs in avians could lead to spillover, causing mammalian (or human) infections and deaths. Currently, no evidence of HPAI H5 transmission has been observed among mammals. However, H5N1 was recently detected in mink farms in the United States and Spain, and more than 50,000 mink were killed to prevent further spread^2–4^. These events provide strong evidence that HPAI H5 viruses can spread rapidly among mammals and that minks may serve as a potential intermediate host to increase the possibility of the H5N1 epidemic in humans. In fact, human infections caused by the HPAI H5 viruses have recently been reported in Ecuador, Cambodia, and Chile (https://www.who.int/emergencies/disease-outbreak-news/item/2023-DON434; https://www.cdc.gov/flu/avianflu/spotlights/2022-2023/chile-first-case-h5n1-addendum.htm).

More concerning evidence has been reported on human infection with the H5N6 subtype virus. From 2014 to 2022, 87 human cases of H5N6 infection were reported (86 in China and one in Laos), with most infections reported in 2021 and 2022 (Fig. 3b). The number of H5N6 infection cases in 2021 and 2022 accounted for 67% of the total number of infections from 2014 to 2022, suggesting that the Chinese government should strengthen protective measures to prevent further spread of the H5N6 virus. Additionally, the first case of H5N8 infection in humans was reported in Russia in 2020^25^. The significant increase in human cases of H5N6 and the emergence of a new human case of H5N8 are alarming signs for human safety.

### Analysis of HPAI H5 HA sequences and assessment of the risk for human infection based on the infection data from 2019 to 2022

In the process of viral infection, HA binds to sialic acid receptors on the cell surface and mediates the fusion of the viral membrane and the host endosomal membrane to deliver viral nucleic acid into the cytoplasm of host cells, thereby playing a key role in the process of infection. HA protein is hydrolyzed to produce HA1 and HA2. HA1 binds to cell receptors via the receptor-binding domain (RBD) and is prone to mutations, while HA2 mediates the membrane fusion process and is relatively conserved. The HA1 of AIVs binds to the α-2,3-sialic acid receptors in avian species, while it binds to the α-2,6-sialic acid receptors in humans. The difference in receptor usage partly prevents the transmission of AIVs from birds to humans. Therefore, we compared HA sequence, HA1 sequence, and RBD key sites of HPAI H5 viruses isolated from avians and humans in recent years to assess the potential risk of human infection.

**Fig. 4:**
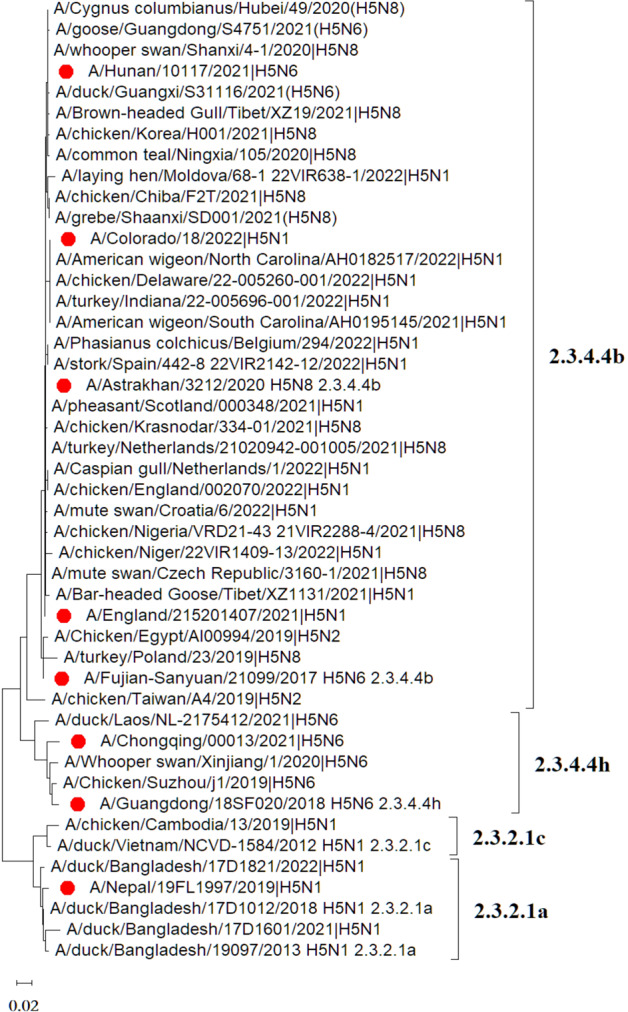
Phylogenetic tree based on HA amino acid sequences of HPAI H5 viruses isolated between 2019 and 2022. These sequences were obtained from GISAID. The human-derived HPAI H5 sequences are marked in red. The phylogenetic tree was drawn with MEGA 11 obtained from https://megasoftware.net/.

The amino acid sequences of HA proteins were derived from GISAID (https://gisaid.org/). The major HPAI H5 HA sequences from avian and human (Fig. 4) belong to subclades 2.3.4.4b (H5N1, H5N6, H5N8), 2.3.4.4h (H5N6) and 2.3.2 (H5N1). Notably, human infection was closely related to the outbreak of AIVs in avians since strains that caused both human and avian infections showed a close evolutionary relationship in all subclades of HPAI viruses, except for 2.3.2.1c. In addition, subclades 2.3.4.4b and 2.3.4.4h mainly broke out in Europe, Africa, Asia, and the Americas, while subclade 2.3.2 has only recently appeared in Egypt, South Asia, and other countries. HA1 amino acid sequences of HPAI H5 were highly homologous with only individual mutations, or even no mutations, for strains from different hosts, subtypes, and regions separated by time (Table 1). The HA1 sequence of human-derived H5N8 strain A/Astrakhan/3212/2020 was used as a reference sequence for comparison with the HA1 sequences of strains derived from humans or birds. It was surprising to find that the HA1 sequence of A/Astrakhan/3212/2020 from humans was identical to that of A/chicken/Kosovo/22-2 22VIR3124-13/2022 from avian. Moreover, only one amino acid (T192I) separated it from another avian-derived HA1 (A/whooper swan/Shanxi/4-1/2020), suggesting that these avian-derived strains hold a high risk for human infection. Most of these site differences in HA1 are located in the RBD region (E130D, A144T, V152L R173Q, T192I, and V214A). Previous studies have performed key amino acid mutations in RBD to analyze the effects on α-2,3/6-sialic acid affinities^26–33^. We collected these key amino acid mutations and compared them with the recent sequences from avian and human sources of the H5N1, H5N6, and H5N8 subtypes (Table 2).

**Table 1:** Differences in HA1 amino acid sequences between HPAI H5 virus derived from humans and avians.

Host	Isolation	Mutation
Human	A/Astrakhan/3212/2020 | H5N8	Reference sequence
Human	A/England/215201407/2021 | H5N1	A144T
	A/Hunan/10117/2021 | H5N6	T192I
	A/Colorado/18/2022 | H5N1	L108M, V214A
Avian	A/Brown-headed Gull/Tibet/XZ19/2021 | H5N8	H277N
	A/chicken/Korea/H001/2021 | H5N8	Q19R
	A/common teal/Ningxia/105/2020 | H5N8	V214I
	A/whooper swan/Shanxi/4-1/2020 | H5N8	T192I
	A/Cygnus columbianus/Hubei/49/2020 | H5N8	T192I
	A/goose/Guangdong/S4751/2021 | H5N6	E130D
	A/American wigeon/North Carolina/AH0182517/2022 | H5N1	L108M, V214A
	A/Phasianus colchicus/Belgium/294/2022 | H5N1	Q19K
	A/stork/Spain/442-8 22VIR2142-12/2022 | H5N1	Q19K
	A/mute swan/Croatia/6/2022 | H5N1	S124N
	A/Caspian gull/Netherlands/1/2022 | H5N1	V152L
	A/chicken/England/002070/2022 | H5N1	V152L
	A/turkey/Netherlands/21020942-001005/2021 | H5N8	No mutation
	A/chicken/Krasnodar/334-01/2021 | H5N8	No mutation
	A/pheasant/Scotland/000348/2021 | H5N1	No mutation
	A/chicken/Nigeria/VRD21-43 21VIR2288-4/2021 | H5N8	R173Q

**Table 2:** Comparison of key amino acid sites in the RBD region of HPAI H5 viruses.

Key amino acid site	133	136	137	138	153	158	160	183	186	190	192	193	196	216	221	225	226	227	228
Avian^a^	L	T/A	S	A	A	N	T	F	N	E	T	L	Q	R	S	G	Q	S	G
Human^a^	V	S	A	V	W	S	A	H	K	D	I	R	H	E	P	D	L	N	S
*H5N8 HA1 from avian and human*																			
A/chicken/Kosovo/22-2 22VIR3124-13/2022^b^	L	S	A	A	W	N	A	H	N	E	T	N	K	K	S	G	Q	R	G
A/whooper swan/Shanxi/4-1/2020H5N8	.	.	.	.	.	.	.	.	.	.	I	.	.	.	.	.	.	.	.
A/chicken/Egypt/Army/1201/2022	R	.	.	.	.	.	.	.	.	.	I	.	.	.	.	.	.	.	.
A/Astrakhan/3212/2020^c^	.	.	.	.	.	.	.	.	.	.	.	.	.	.	.	.	.	.	.
*H5N6 HA1 from avian and human*																			
A/duck/Hunan/S40199/2021	.	.	.	.	.	.	.	.	.	.	.	.	.	.	.	.	.	.	.
A/duck/Yunnan/S4318/2021	.	.	.	.	.	.	.	.	.	.	I	.	.	.	.	.	.	.	.
A/duck/Zhejiang/S4854/2021	.	.	.	.	.	.	.	.	.	.	.	D	.	.	.	.	.	.	.
A/Hunan/09285/2021	.	.	.	.	.	.	.	.	.	.	I	.	.	.	.	.	L	.	.
A/Chongqing/02/2021	.	.	.	.	.	.	.	.	.	.	I	.	.	.	.	.	X	.	.
A/Guangdong/18SF020/2018	.	.	.	.	.	.	.	.	.	.	.	D	.	.	.	.	.	.	.
A/Jiangsu/1/2020	.	.	.	.	.	.	.	.	.	.	.	K	.	.	.	.	.	G	.
A/Chongqing/00013/2021	.	.	.	.	.	.	.	.	.	.	.	V	.	.	.	.	.	.	.
A/Fujian-Sanyuan/21099/2017	.	.	T	.	.	.	.	.	.	.	T	.	.	.	.	.	.	.	.
*H5N1 HA1 from avian and human*																			
A/duck/Guizhou/S1321/2022	.	.	.	.	.	.	.	.	.	.	V	.	.	.	.	.	.	.	.
A/laying hen/Moldova/68-2 22VIR638-2/2022	.	.	.	.	.	.	.	.	.	.	.	.	.	.	.	.	.	.	.
A/duck/Bangladesh/17D1821/2022	.	.	.	.	.	D	.	.	.	.	.	R	Q	.	.	.	.	S	.
A/chicken/Hong Kong/AP156/2008	.	.	.	.	.	.	T	.	.	.	.	R	Q	.	.	.	.	S	.
A/duck/Egypt/D1Br12/2007	S	.	S	.	.	.	T	.	.	.	.	M	Q	.	.	.	.	S	.
A/Nepal/19FL1997/2019	.	.	.	.	.	.	.	.	.	.	.	R	Q	.	.	.	.	N	.
A/Laos/2121/2020	.	.	.	.	.	D	.	.	X	.	.	R	Q	R	P	.	.	S	.
A/England/215201407/2021	.	.	.	.	.	.	.	.	.	.	.	.	.	.	.	.	.	.	.
A/Anhui/1/2005	S	.	S	.	.	.	T	.	.	.	.	K	Q	.	.	.	.	S	.
A/Thailand/1(KAN-1)/2004	.	.	S	.	.	.	T	.	.	.	.	K	Q	R	.	.	.	S	.
A/Vietnam/1203/2004	S	.	S	.	.	D	.	.	.	.	.	R	Q	.	.	.	.	S	.
A/Indonesia/05/2005	.	.	S	.	.	.	T	.	.	.	.	K	Q	R	.	.	.	S	.

Sequence data were downloaded from https://legacy.fludb.org/brc/home.spg?decorator=influenza.

^a^Typical amino acids at the indicated positions in avian- or human-susceptible H5 viruses.

^b^Reference sequence.

^c^HPAI viruses isolated from humans are underlined.

**Fig. 5:**
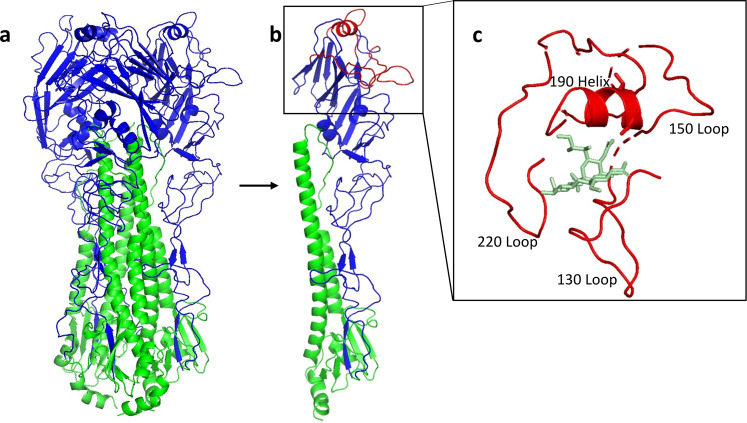
Structure of HA protein derived from HPAI H5 virus. **a** Trimeric HA is shown in cartoon representation with HA1 displayed in blue and HA2 in green. **b** Critical domains, including three loops and one helix, in RBD are shown in red in a single HA molecule. **c** Red domains in RBD (130-loop, 150-loop, 190-helix, and 220-loop) from **b** were enlarged and displayed with a sialic acid molecule (light green) in the groove. HA sequence of A/Astrakhan/3212/2020 was modeled by SWISS-MODEL and drawn by PyMOL software obtained from https://pymol.org/2/. The PDB number for the α-2,6-sialic acid molecule is 5E35.

RBD is located in the head of HA1 and contains 190-helix, 130-loop, 150-loop, 220-loop, and other amino acid residues^26,34,35^ (Fig. 5). Yang et al.^36^ found that the introduction of S137A and T192I mutations in the RBD of A/Thailand/KAN 1/2004 endowed this Avian strain with the ability to bind with α-2,6-sialic acid receptors present in humans. In our selected sequences, the 137A and 192I sites were found to be present in both human- and avian-derived H5N8 and H5N6 strains, indicating that they are key sites for cross-species transmission. In addition, although 192T exists in A/Astrakhan/3212/2020 (H5N8) and A/Fujian-Sanyuan/21099/2017 (H5N6), these strains still retain the ability to infect humans. This indicates that a single-site mutation (S137A/T) may also change the receptor-binding ability of AIVs. It has been reported that some mutation sites, such as the K193R mutation in the A/Vietnam/1203/2004 strain^37^, the Q196H mutation in the A/duck/Egypt/ D1Br12/2007 strain^38^, and the Q226L, S227N, and G228S mutations in the A/Indonesia/05/2005 strain^39^, can enhance the ability of stains to utilize the α-2,6-sialic acid receptor. In our selected sequences, the 193R site is present in both human- and avian-derived H5N1 strains, indicating that these H5N1 strains may have already gained the ability to utilize α-2,6-sialic acid receptors.

### Antiviral therapy for influenza

Some small-molecule compounds have been developed for the treatment of influenza viruses. These compounds target various stages of the viral life cycle, e.g., virus adsorption, fusion, nucleic acid release, nucleic acid replication, and virus budding. HA protein inhibitors block virus adsorption or fusion, which can be divided into HA1 and HA2 inhibitors. HA1 inhibitors, such as Dextran sulfate and DSA181^40^, block the binding of HA1 to receptors on the cell surface. Meanwhile, HA2 inhibitors like arbidol^41^ and BMY-27709^42^ block virus entry by preventing HA2-mediated membrane fusion. In addition, Basu et al. identified two small-molecule compounds, MBX2329 and MBX2546, which were able to bind to the stem region of the HA trimer and inhibit HA-mediated fusion^43^. The fusion process of the influenza virus also depends on endosomal acidification and a series of host enzymes, like proteases. Therefore, inhibitors of these host enzymes have also been developed as anti-influenza drugs, such as bafilomycin A1^44^ and aprotinin^45^. After membrane fusion, viral RNA enters the host cell through the M2 ion channel. M2 inhibitors like amantadine and rimantadine, which block ion channel activity, were developed to prevent the release of the viral genome into the cytoplasm. M2 inhibitors are effective for the influenza A virus but not for the influenza B virus because of its lack of M2 protein. It has been reported that S31N mutation is the main culprit causing resistance to M2 inhibitors, thus accounting for 92% of drug-resistant strains in the United States^46^. Consequently, M2 inhibitors are currently not recommended for treatment. NA protein is related to the maturation and release of viruses, and it plays an important role in regulating receptor binding and virus budding. NA inhibitors, such as oseltamivir, zanamivir, and peramivir, can effectively inhibit the release of progeny viruses from infected cells^47,48^. However, amino acid mutations in the NA protein, e.g., E119A, H274Y, and N294S, usually lead to resistance to NA inhibitors^49,50^. Furthermore, viral nucleic acid replication inhibitors include PB2 inhibitors (VX787^51^), PA inhibitors (flutamide^52^ and Baloxavir^53^), RNA-dependent RNA polymerase (RdRp) inhibitors (Favipiravir^54^), and NP inhibitors (nucleolin^55^).

In addition, some monoclonal antibodies (Mab) have been developed and are highly anticipated for post-exposure prophylaxis and clinical treatment. For example, a novel humanized Mab 8A^56^ neutralized H5N1 by binding to two types of epitopes on HA. Li et al. described a chimeric Mab, termed C12H5, which could neutralize representative strains of H1N1 circulating from 1991 to the present; it could even cross-neutralize H5N1^57^. It has been reported that neutralizing antibodies against HA, isolated from volunteers vaccinated with seasonal influenza vaccines, could protect mice from H1N1 and H3N2 viruses in vivo^58^. Recently, the FDA confirmed that humanized polyclonal antibody SAB-176 could recognize multiple epitopes and provide protection against multiple influenza virus strains (https://ir.sab.bio/static-files/b332c893-5795-4d05-af96-a9ebcd917f24). A phase 2b clinical trial is about to be launched in patients with high-risk severe diseases. Some polypeptide drugs, such as EB-peptide^59^, iHA^60^, FluPep^61^, NDFRSKT^62^, P1^63^, and P9R^64^, have also been developed against influenza viruses. However, the accumulation of mutations in AIVs still increases the probability of immune evasion^65,66^. Therefore, updating existing antiviral drugs cannot keep pace with the continuous variation of AIVs. This calls for new antiviral strategies, such as drugs and therapeutic Mabs targeting more conserved viral epitopes or cytokines, or immunomodulatory drugs, in response to emerging strains with epidemic potential^43,67^.

### Development of H5-specific influenza vaccines

Currently, the main types of avian influenza vaccines include inactivated recombinant vaccines, subunit vaccines, viral vector vaccines, and DNA vaccines. Inactivated vaccines were previously the primary means of preventing influenza and were mainly prepared from low pathogenicity strains isolated from farms^68^. However, traditional inactivated vaccines are not conducive to vaccine production owing to such defects as dependence on embryo culture and low virus titer. At present, the vaccines used for H5 and H7 avian influenza in China are mainly recombinant inactivated vaccines. These vaccines are prepared by co-transfecting Vero cells with the viral RNA expression plasmid of HA and NA genes of the current epidemic strains and six internal genes (PB2, PB1, PA, NP, M, and NS) of PR8 (A/Puerto Rico/8/1934), together with four PR8 protein expression plasmids (PB2, PB1, PA, and NP)^69^. The basic terminal sequence R/KRRKR of HA from the HPAI virus was modified to RETR, endowing the recombinant virus with both the epitope of the pandemic strain and the high-titer characteristics of PR8 chicken embryo adaptation. The team led by Dr. Hualan Chen in China has developed a series of recombinant vaccines for the prevention of HPAI H5, among which Re-13 (A/duck/Fujian/S1424/2020 H5N6 2.3.4.4h) and Re-14 (A/whooper_ swan/Shanxi/4-1/2020 H5N8 2.3.4.4b) were developed in 2022^70^. According to Chen et al.^71^, the H5N1 AIV strain bearing the subclade 2.3.4.4b HA gene was isolated from China in 2021–2022 and exhibited antigenic sites similar to those of H5-Re14. Since this type of recombinant vaccine is widely used in China, it plays a crucial role in the prevention and control of AIVs. WHO updated its AIV strain recommendations in 2022 and selected A/Astrakhan/3212/2020 H5N8 2.3.4.4b, A/Guangdong/18SF020/2018-like H5N6 2.3.4.4h and A/Fujian-Sanyuan /21099/2017-like H5N6 2.3.4.4b as candidate vaccine strains (https://www.who.int/teams/global-influenza-programme/vaccines/who-recommendations).

### Development of universal influenza vaccines

As AIV is a single-stranded RNA virus, its nucleic acid sequence is prone to mutation, thereby reducing the protective efficacy of the vaccine over time. Although it is possible to predict the next dominant strain for vaccine strain selection, production, and distribution, the circulating strain may further mutate, resulting in a decrease in vaccine protection efficiency. Therefore, it is necessary to develop universal influenza vaccines that target more conservative epitopes to counter potential antigenic drift or shift in AIVs. Accordingly, scientists have focused on several common targets for the development of universal influenza vaccines, including the conserved stalk domain of HA protein, the conserved regions of NA protein, the ectodomain of M2 ion channel (M2e), and the internal proteins, nucleoprotein (NP) and matrix protein 1 (M1). The aim is to expand existing immune memory response by multiple immunizations in order to produce the widest range of protective antibodies against different subtypes of influenza virus^72^.

Effective humoral heterosubtypic immunity is rare, mainly based on antibodies targeting the HA stalk domain^73,74^. As mentioned, the RBD region in the head of HA protein is prone to mutations leading to viral immune evasion, while the HA stalk domain is rarely exposed to neutralizing antibodies, thus facing less selection pressure from the host immune system. As a result, the HA stalk domain is highly conserved in AIVs, making it an attractive target for universal vaccine design. A strategy for inducing high levels of stalk-reactive antibodies is based on chimeric HAs (cHAs), which combine exogenous head domains with conserved stalk domains. The cHAs with different head domains have been used in sequential vaccination programs to break the immunodominance of the head domain of HA and induce high titers of stalk-reactive antibodies^75^. However, the vaccine targeting the conserved stalk domain of HA can only produce cross-protection that occurs between strains within the same subtype or multiple subtypes of the same group, making it difficult to induce broadly reactive antibodies against influenza viruses across different groups. However, the construction of chimeric HA stalk domain with other conserved antigens, such as M2e, could improve cross-protection against multiple AIVs from different groups and improve the broad protection of universal influenza vaccines^76^. Chen et al. ^77^ have reported that influenza virus infection induces high titers of NA-reactive antibodies, which effectively inhibit the enzymatic activity of NA and provide robust prophylactic protection against avian H5N1 viruses in vivo. This observation suggests that some conserved regions in NA recognized by NA-reactive antibodies could be incorporated into influenza vaccines to elicit durable and broad protection against divergent influenza strains.

Emerging vaccine platforms can help trigger a better immune response than that induced by traditional influenza vaccines. For example, virus-like particles (VLPs) can present natural conformational antigens, stimulate the immune system through a virus-like pathway, and efficiently induce immune protection. An H5N1 VLP-based vaccine, designed with computationally optimized broadly cross-reactive antigen (COBRA), elicits broadly reactive antibodies in mice and ferrets. Therefore, this strategy is potentially paradigm-shifting for H5 universal influenza vaccines^78^. A candidate universal influenza vaccine, which uses M2e-based VLP to present M2e, has been shown to protect mice from homosubtypic and heterosubtypic AIVs^79^. In addition, nanoparticle platforms have been used to develop universal influenza vaccines owing to their dominance in expressing antigens at high densities and providing adjuvant-like functions. For example, the OVX836 vaccine is based on oligomerized nanoparticles (NPs) that can induce humoral and cellular immunity in mice and ferrets, thereby providing protection against influenza A and B^80,81^. A ‘mosaic’ quadrivalent influenza vaccine based on two-component nanoparticle immunogens not only showed better protective antibody response than the 2017–2018 quadrivalent influenza vaccine (QIV) but also triggered heterosubtypic antibody response and protective immunity in several animal models^82^. Viral vector-based vaccines can be delivered through both systemic or mucosal routes to trigger strong humoral and cellular immunity. An adenovirus vector-based H5N1 conserved multi-epitope influenza vaccine showed broad immune protection against H5, H7, and H9 influenza viruses in mice^83^. The nucleic acid platform includes DNA- and mRNA-based vaccines, which can respond quickly to emerging outbreaks. Based on their novel contribution to the coronavirus disease 2019 (COVID-19) pandemic, mRNA-based vaccines have become the focus of new vaccine technologies. Freyn et al. ^84^ demonstrated the broad protective effect of nucleoside-modified mRNA-LNP vaccines based on conserved antigens (HA, NA, NP, and M2) against influenza virus challenge in mice. Koen et al. ^85^ evaluated heterosubtypic protection from a nucleoside-modified mRNA vaccine that encodes the conserved NP, M1, and PB1 (polymerase basic protein 1) of one H1N1 strain. This vaccine induced a broadly reactive T-cell response in ferrets. Recently, Arevalo et al.^86^ developed an mRNA-LNP vaccine encoding HA from 20 known influenza A and B virus subtypes, and it triggered high levels of cross-reactivity and subtype-specific antibodies in mice and ferrets. This is a new antigen design concept for developing a universal influenza vaccine.

In addition, new vaccine adjuvants support ideas for universal vaccine design. Appropriate vaccine adjuvants can improve immunogenicity, regulate immune response types, and even enhance the universality of vaccine protection^87,88^. Only 6 new adjuvants have been approved by the FDA in the past century, including MF59, AS04, AS03, AS01, CpG1018, and Matrix-M adjuvants for emergency use in COVID-19. MF59 and AS03 have improved the protective efficiency of influenza vaccines^73,89^. More prominently, the quadrivalent influenza nanoparticle vaccine (qNIV) with Matrix-M has been shown to enhance antigen presentation, expand the antibody epitope library, boost cross-neutralizing antibody responses, and improve the induction of potent CD4^+^ and CD8^+^ T cell responses in a variety of cells^90^. It has now successfully completed key phase III trials. Compared with adjuvant-free vaccines, influenza vaccines with adjuvants have shown higher immunogenicity and effects on heterologous strains. The 2′,3′-cyclic guanosine monophosphate-adenosine monophosphate (cGAMP) is an effective natural agonist of stimulator of interferon genes (STING), which induces type I interferon (IFN-I) response and proinflammatory cytokine production by activating STING^91,92^. Wang et al.^93^ demonstrated intranasal immunization with the PS-cGAMP-adjuvanted inactivated H1N1 vaccine, which triggered a strong protective effect against different subtypes of influenza (H3N2, H5N1, and H7N9) in mice. During the COVID-19 pandemic, the emerging non-nucleotide small-molecule STING agonist CF501 showed higher protective efficacy compared to the cGAMP-adjuvanted vaccine, suggesting that CF501 can also be used as an adjuvant to boost the original vaccine for effective, extensive and long-term immune protection^94^.

In this article, we have analyzed the global epidemic of HPAI H5 and revealed that the number of infections has risen significantly in recent years. Furthermore, it has been observed that the dominant HPAI virus worldwide rapidly changed from H5N8 to H5N1 in 2022. According to the sequence alignment analysis of HA1, we found that the HA1 sequences of strains isolated from avians and humans were highly homologous or even identical, suggesting that the existing AIVs strains circulating in birds may infect humans. Amino acids 137A, 192I, and 193R in the RBD of HA are key sites that exist in both avian and human source sequences. These sites enable the current HPAI H5 strains to bind α-2,6-sialic acid receptors in humans, indicating that the mutated HPAI H5 viruses may have jumped from birds to mammals and that such spillover may cause human infection.

It should be mentioned that receptor affinity is not the only factor affecting the transmission of AIVs in humans. In the process of viral infection, HA mediates membrane fusion between the virus and host cells^95^. Next, nucleic acid is released with the assistance of the M protein and enters the nucleus to complete viral replication in the presence of viral polymerases PA, PB1, and PB2. Finally, the progeny virus is released from infected cells with the assistance of the NA protein. Many HPAI H5 viruses can enter host cells, but they cannot replicate successfully owing to the difference in amino acids at position 627 of PB2 protein, namely glutamic acid in AIVs and lysine in human influenza virus^96^. Hence, mutations in the RBD domain may only affect receptor binding and cell entry of AIVs, while replication efficiency of the virus in cells must be assisted by other viral proteins, such as PA, PB1, PB2, and NA, to gain successful cross-species transmission^97^. Therefore, mutations in these proteins and homologous recombination between strains deserve more attention.

Nowadays, the HPAI H5 virus belonging to the 2.3.4.4b subclade is widespread among wild birds and poultry worldwide, resulting in significant economic losses. The prevention and control strategy for the HPAI H5 virus in Europe and North America mainly relies on culling, while the strategy in China is “vaccine and culling”. The latter strategy did reduce HPAI H5 virus infections in avians in China (Fig. 1a)^25^. In addition, after vaccination of the H5/H7 vaccine in poultry, the isolation of H7N9 strains in China decreased by 93.3%, which largely prevented the prevalence of H7N9 among poultry^98^. The transmission modes of HPAI H5 among wild birds, poultry, and mammals also deserve more attention. Wildfowl is the natural host of the HPAI H5 virus, and the virus usually replicates in their intestines and respiratory tract. Nine major routes have been identified for migration across the world, increasing the likelihood of AIV infection in poultry and mammals^99,100^. Therefore, understanding the temporospatial characteristics and as well as environmental factors of HPAI H5 outbreaks is helpful for establishing an effective prevention and control system^101^. It is widely accepted that AIVs only infect mammals through avian transmission and that no reports have so far indicated its spread among mammals. Nonetheless, the recent spread of the H5N1 virus in mink has sounded the alarm for human safety^2–4^. Prevention should be emphasized in virus-susceptible areas, and measures should be taken to reduce human exposure to birds and mammals in order to minimize the risk of zoonotic infections. Protective measures and preventive vaccination should be taken seriously for populations susceptible to occupational hazards. Finally, real-time virus monitoring and rapid data sharing are crucial for assessing the risk of cross-species transmission of HPAI H5 and implementing effective prevention and control measures. The antigenic drift of the current epidemic strains should be monitored, and it should be determined whether the existing vaccines still have protective effects. Furthermore, H5-specific vaccines need to be developed, and the team led by Hualan Chen in China, whose work was noted above, serves as a model in this regard^71,102,103^.

While small-molecule compounds, peptides, and antibodies have been developed for influenza antiviral therapy, the constant mutation of the virus and its ability to evade immune response confound these efforts. Therefore, drugs and vaccines must be regularly updated to address the emergence of new strains. In response, scientists are trying different methods to develop universal vaccines against multiple subtypes of influenza viruses. HA is the main immunogen for vaccine design and mainly induces antibodies against the RBD region at the spherical head of HA, which is also highly prone to mutation. However, some cross-protective antibodies against highly conserved HA stalk may also be induced^104^. Emerging vaccine platforms and new vaccine adjuvants also provide pathways toward improving vaccine efficacy. Although not emphasized in this review, the potential of cross-reactive T cell-based responses for influenza vaccine design cannot be ignored. Currently, avian influenza vaccines are mandatory for poultry immunization. However, they are not included in routine human immunization but are only used as a preventive vaccination strategy during emergencies. HPAI H5 viruses are circulating in birds and have even caused outbreaks in mammals in recent years, thus raising concerns about HPAI H5 infections in humans. Heterologous prime–boost immunization strategies against H5N1 could induce broader cross-clade antibody responses. It is also worth considering priming with a universal vaccine and boosting with a specific vaccine against the current pandemic strain.
